# Classification of Kinematic and Electromyographic Signals Associated with Pathological Tremor Using Machine and Deep Learning

**DOI:** 10.3390/e25010114

**Published:** 2023-01-05

**Authors:** Alejandro Pascual-Valdunciel, Víctor Lopo-Martínez, Alberto J. Beltrán-Carrero, Rafael Sendra-Arranz, Miguel González-Sánchez, Javier Ricardo Pérez-Sánchez, Francisco Grandas, Dario Farina, José L. Pons, Filipe Oliveira Barroso, Álvaro Gutiérrez

**Affiliations:** 1 E.T.S. Ingenieros de Telecomunicación, Universidad Politécnica de Madrid, 28040 Madrid, Spain; 2 Neural Rehabilitation Group, Cajal Institute, Spanish National Research Council (CSIC), 28002 Madrid, Spain; 3 Department of Bioengineering, Imperial College London, London SW7 2AZ, UK; 4 Movement Disorders Unit, Department of Neurology, Hospital General Universitario Gregorio Marañón, 28007 Madrid, Spain; 5 Department of Medicine, Universidad Complutense, 28040 Madrid, Spain; 6 Legs & Walking AbilityLab, Shirley Ryan AbilityLab, Chicago, IL 60611, USA; 7 Department of PM&R, Feinberg School of Medicine, Northwestern University, Evanston, IL 60208, USA; 8 Department of Biomedical Engineering and Mechanical Engineering, McCormick School of Engineering, Northwestern University, Evanston, IL 60208, USA

**Keywords:** machine learning, tremor, LSTM, electrical stimulation

## Abstract

Peripheral Electrical Stimulation (PES) of afferent pathways has received increased interest as a solution to reduce pathological tremors with minimal side effects. Closed-loop PES systems might present some advantages in reducing tremors, but further developments are required in order to reliably detect pathological tremors to accurately enable the stimulation only if a tremor is present. This study explores different machine learning (K-Nearest Neighbors, Random Forest and Support Vector Machines) and deep learning (Long Short-Term Memory neural networks) models in order to provide a binary (*Tremor*; *No Tremor*) classification of kinematic (angle displacement) and electromyography (EMG) signals recorded from patients diagnosed with essential tremors and healthy subjects. Three types of signal sequences without any feature extraction were used as inputs for the classifiers: kinematics (wrist flexion–extension angle), raw EMG and EMG envelopes from wrist flexor and extensor muscles. All the models showed high classification scores (*Tremor* vs. *No Tremor*) for the different input data modalities, ranging from 0.8 to 0.99 for the f_1_ score. The LSTM models achieved 0.98 f_1_ scores for the classification of raw EMG signals, showing high potential to detect tremors without any processed features or preliminary information. These models may be explored in real-time closed-loop PES strategies to detect tremors and enable stimulation with minimal signal processing steps.

## 1. Introduction

Neural disorders are the leading cause of disability worldwide. In particular, pathological tremors are one of the most common motor disorders in the adult population and can severely hamper the execution of activities of daily living (ADLs) [1]. A pathological tremor refers to the oscillatory and involuntary movements of one or more body parts in the frequency band above 4 Hz [2]. For some patients, the amplitude and constant presence of the tremor oscillations affects the performance of ADLs [3] and the tremor becomes a disabling condition, not only impacting the motor function but also the mental health of the patients as they suffer comorbidities, such as depression or stress. It is worth noting pathological tremors differs from physiological tremors, which are also described as involuntary limb oscillations in the frequency band between 8 Hz and 12 Hz [4]. Physiological tremors are observed in healthy individuals with different features. However, they do not compromise ADLs, their amplitude is reduced and their frequency is typically higher compared with pathological tremors [5].

Essential Tremor (ET) is the main clinical diagnosis leading to pathological tremors, with an estimated incidence of around 4% to 5% of the population above 65 years old [6]. An ET diagnosis is purely clinical, as upper limb action in the frequency band between 4 Hz and 12 Hz is the main clinical manifestation, and the disease etiology and pathophysiology are not completely understood [7,8]. Besides its causes, the major challenge in ET is the absence of a cure, since current treatments are limited to symptomatic relief, i.e., tremor intensity reduction [9]. The first line of treatment is pharmacotherapy, but approximately 50% of the patients do not benefit from this because of its efficacy or its associated adverse effects [9]. Neurosurgical interventions, such as Deep Brain Stimulation or High-Intensity Focused Ultrasound, are the most effective solutions for the most severe tremor cases [10]. However, there is a limited number of patients who are eligible for these procedures due to the surgery-related risks, elevated costs and reluctance due to the potential adverse effects [6,11]. Therefore, there is an increasing interest in alternative tremor management solutions which are less invasive and overcome the side effects of standard treatments with similar efficacy [12,13].

Peripheral Electrical Stimulation (PES) of afferent pathways (stimulation below motor threshold) is emerging as a potential solution to reduce pathological tremors with potentially minimal side effects [14]. PES below motor threshold delivers electrical currents that activate the afferent or sensory pathways which are hypothesized to disrupt tremors at different sites of the central nervous system [15]. Some studies have gathered evidence about the relevance of synchronizing the stimulation with physiological events, such as the tremor phase or frequency, in order to promote neural adaptations in the spinal cord or brain circuits which prevent the tremor input from reaching the muscles [16]. Thus, the development of stimulation strategies that integrate bio-signals, such as kinematic or electromyography (EMG) measurements, into closed-loop control systems in order to adapt the stimulation to the tremor features might be a cornerstone for the optimization of PES-based tremor reduction therapies. The application of stimulation while a tremor is present and timed with the tremor phase might present some benefits: the maximization of neural plasticity induction according to paired associative stimulation protocols [17]; a reduction in the stimulation dose and a likely reduction in the development of tolerance [16]; or the minimization of interference with voluntary movements [18].

So far, very few studies have tested the closed-loop PES of afferent pathways in order to reduce tremors, and these studies have used algorithms based on traditional signal processing methods [16]. Some of the closed-loop strategies enable stimulation only if tremor is detected using EMG or kinematic signals measured from the target muscle or joint. Such is the case for the out-of-phase and Selective and Adaptive Timely Stimulation (SATS) strategies, which use sequential recording and stimulation windows [19]. During a recording window (where no stimulation is applied) the envelope in the tremor band is extracted by means of rectification and band-pass filtering or the Hilbert Transform application of the raw signals [20]. Then, the tremor frequency and period are estimated by identifying the local maxima (tremor peaks) in the envelope, which are above a heuristic threshold. If a tremor is detected, the stimulation window is enabled, and the stimulation is delivered based on the next predicted tremor cycle (out-of-phase) or real-time tremor estimation (SATS) [15]. Other groups have proposed tremor detection methods based on computing the power spectral density (PSD) of the envelope of the EMG signal [21,22]. In this case, the power peak in the tremor band is identified and compared against a threshold that has been previously empirically estimated with data recorded from healthy subjects without tremors.

A common limitation of tremor detection methods is the use of user-defined thresholds to detect tremors either based on the time or frequency features of the signals, as this procedure requires preliminary data and operator experience. The application of machine learning techniques to classify or predict pathological signals may overcome the limitations of traditional approaches. In the literature, there are numerous approaches to quantify and monitor pathological tremors using machine learning techniques or EMG measurements [23,24]. Alves et al. [25] explored the classification of hand parkinsonian tremors based on machine learning methods (SVM, KNN and Random Forest, among others) using 272 features extracted from kinematics data measured with Inertial Measurement Units (IMUs). Jeon et al. [26] proposed the classification of tremor severity from 15 kinematic features using several machine learning algorithms (SVM and KNN) in order to score parkinsonian tremor severity which matched the Parkinson’s Disease rating scale (UPDRS). These and other implementations use the extraction and identification of features as a preliminary step to perform classification tasks [27]. In the application of closed-loop PES, minimizing pre-processing stages is a desirable characteristic as the methods must be applied continuously in real-time and are likely embedded in wearable systems.

The application of Artificial Neural Networks (ANNs) and deep learning models to model pathological tremors has gained interest in recent years [28]. The higher complexity of some of these models, such as auto-encoders, Recurrent Neural Networks or Long Short-Term Memory (LSTM) neural networks, allows the identification of temporal patterns in a time series [29]. However, whether the increase in the complexity of these models translates into more accurate outcomes compared to traditional machine learning approaches is yet to be proven [30].

Nonetheless, tremorgenic signals, recorded either from kinematics or EMG, arise from spatial and temporal mechanisms containing linear and non-linear information. Previous studies have provided entropy measures that show evidence about disorder in the system [31]. Moreover, some other studies provide an automatic analysis of the characterization of ET based on the linear and non-linear features of digital drawings [32].

The main goal of this study was to develop a tremor detection method based on kinematic and EMG signals. The performance of the binary tremor classification based on traditional machine learning techniques (SVM, KNN and Random Forest) and deep learning models (Long Short-Term Memory networks) was assessed using kinematic and EMG signals recorded from healthy subjects and ET patients. We hypothesized that pre-processed datasets would lead to higher prediction scores, and that the LSTM classifiers would outperform the classification results achieved by traditional models. The models provided here could be integrated into closed-loop PES tremor reduction strategies.

## 2. Materials and Methods

### 2.1. Participants and Data Acquisition

Twelve patients diagnosed with ET from Gregorio Marañón Hospital (Madrid, Spain) and eleven healthy subjects were recruited for this study. For the ET patients, the presence of postural tremors and predominant wrist flexion–extension tremors, and the absence of additional neuromuscular disorders were conditions considered in the inclusion criteria. All the participants signed a consent form, and all the procedures were approved by the hospital Ethics Committee.

**Figure 1:**
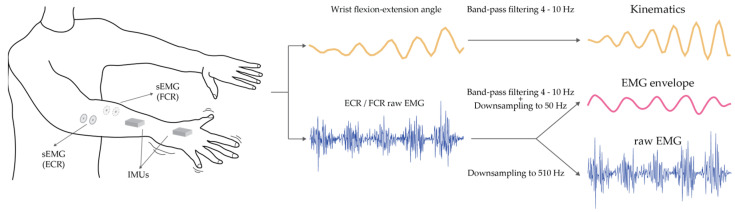
Illustration of the data recording setup (**left**) and pre-processing steps (**right**) followed in order to obtain the three different input datasets. On the left, the sEMG bipolar electrodes were placed over the wrist flexor and extensor muscles; two IMUs were placed on the dorsal side of the hand and the forearm.

Kinematic and EMG signals were acquired while the patients were asked to maintain a posture that facilitated the appearance of tremors for 60 s: with both arms stretched out in front and pronated (see Figure 1), or with both arms raised, elbows flexed and fingers pointing to each other at the face level [33]. The healthy subjects were asked to replicate the same postures. The acquisition of kinematic signals was performed using a motion capture system with a set of two inertial measurement sensors (IMUs, Technaid S.L, Spain) that were placed on the patients’ hand and forearm affected by tremors, or on the dominant side for the healthy subjects [34]. The IMU system provided orientation data in the form of quaternions sampled at 50 Hz. Surface EMG (sEMG) signals were acquired at 2042 Hz with a bio-signal amplifier (Quattrocento, OT Bioelettronica, Italy) using bipolar electrodes placed on the Flexor Carpi Radialis (FCR) and Extensor Carpi Radialis (ECR) muscles. When the healthy subjects held the postural task, the muscle activity of ECR and FCR muscles was close to baseline activity. Hence, additional EMG data were acquired while they were asked to perform an isometric wrist flexion contraction equivalent to 10% of their maximum voluntary contraction [35].

### 2.2. Data Pre-Processing

#### 2.2.1. Kinematics

The raw quaternions were converted into Euler angles and the wrist flexion–extension angle was computed and segmented in 1-s windows. The classification task required the labelling of the kinematic signals into two classes: *Tremor* and *No Tremor*. Following previous implementations, this task was performed based on the spectral power of the signals in the tremor band. The signals were band-pass filtered (2nd order zero-lag Butterworth) between 4 Hz and 10 Hz in order to isolate the component related to the pathological tremor. Furthermore, the PSD was computed for the 1-s window, and the maximum value in the tremor band [4, 10] Hz was identified [36]. Then, the global threshold used to differentiate data samples of patients presenting tremor with those who do not was heuristically defined based on the spectral values. This approach was followed for two main reasons: previous studies have used the PSD as an objective classification for tremors [22]; and if a patient is diagnosed with ET, not all the 1-s signals extracted during tremor recordings must show tremorgenic activity; thus, the classification errors due to mislabeled data are minimized.

The whole dataset was normalized between 0 and 1 (the amplitude of all the signals recorded from all the subjects were mapped in the range [0, 1]). A total of 18,000 s of kinematics data were selected for the final dataset, including balanced subsets of classes: 9000 instances were labeled as *Tremor* and 9000 as *No Tremor*. Then, the kinematics dataset was divided into training (70% of samples) and test sets (30%) for the machine learning models, and training (70%), validation (15%) and test sets (15%) for the LSTM models.

#### 2.2.2. Electromyography

The EMG signals were firstly visually inspected and the recordings with low signal to noise ratios (SNRs) or artifacts were discarded as they did not satisfy the minimum quality requisites. The signals were downsampled from 2042 Hz to 510 Hz in order to reduce the computational cost related to the training and classification stages, and were segmented in 1-s windows (see Figure 1) [37]. This process does not imply the loss of relevant information for bipolar surface EMG signals [38].

In order to explore the capabilities of the models to classify minimally pre-processed signals, two different datasets were created from the EMG signals acquired. The first dataset comprised of the downsampled raw EMG signals, and the second dataset was created from the envelope of the EMG signals in the tremor band. In order to extract the envelope, the raw signals were rectified and band-pass filtered between 4 Hz and 10 Hz (2nd order zero-lag Butterworth). Then, the EMG envelopes were downsampled to 50 Hz as the tremor component was preserved. At this point, either the raw EMG or EMG envelope datasets were normalized between 0 and 1 [39].

Following the same procedure as for the kinematics dataset, data labelling (*Tremor* and *No Tremor*) was performed by identifying the heuristic threshold for the PSD computed for the EMG envelopes in the tremor band. The final datasets for the raw and envelope EMG signals consisted of 8000 samples each, including 4000 samples for each of the two classes (Tremor and No Tremor). Finally, the EMG datasets were divided into training (70% of samples) and test sets (30%) for the machine learning models, and training (70%), validation (15%) and test sets (15%) for the LSTM models.

### 2.3. Classication Algortihms

All the pre-processing and classification algorithms were implemented using Python [40]. In particular, the packages *scikit-learn* were implemented for the machine learning models [41], and *PyTorch* [42] for the LSTM neural networks.

#### 2.3.1. Machine Learning Classifiers

Different machine learning algorithms that are widely used were selected to solve the problem of tremor binary classification. In order to find the optimal hyper-parameters for each of the machine learning methods, K-fold cross-validation (k = 10) was applied.

- K-nearest neighbors (KNN). The fixed parameters selected included the algorithm (Ball tree) and weights (Distance). The variable parameters were the number of neighbors, the leaf size and the distance algorithm [43].
- Support Vector Machine (SVM). The selected kernel was the Radial Basis Function (RBF), whereas the hyperparameters *c* and γ were explored in the validation process [44].
- Random Forest (RF). The criterion function (Entropy) and the minimum number of samples per leaf were fixed, whereas the number of trees and the maximum number of features were varied across the cross-validation method [45].

#### 2.3.2. LSTM Classifier

**Figure 2:**
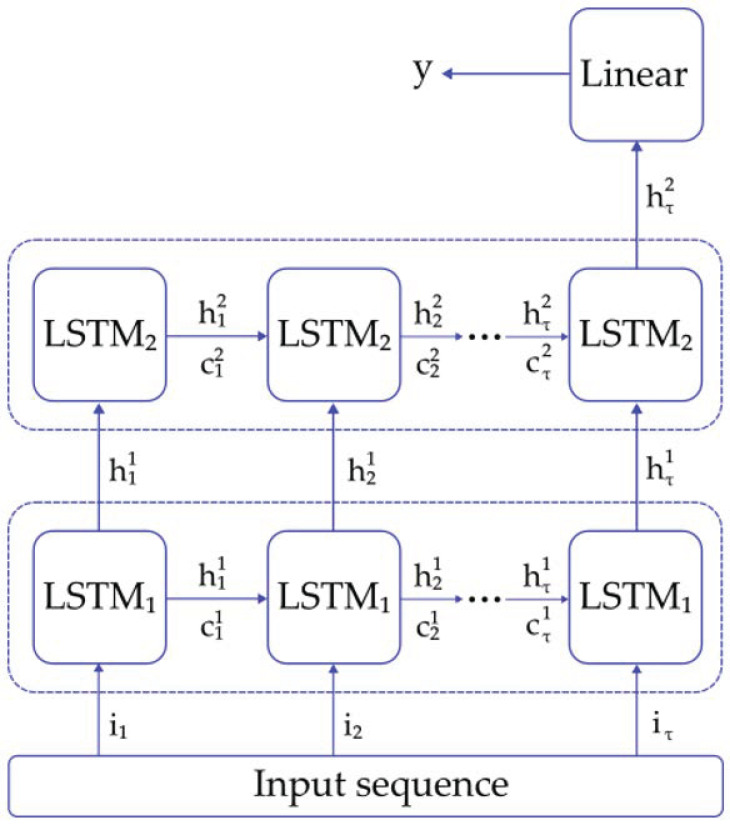
LSTM architecture used for the classification of pathological tremors.

The LSTM neural network model used for classification is shown in Figure 2. The architecture selected consists of three layers: two LSTM layers and a final linear layer, which was a perceptron [46]. The inputs for the first LSTM layer were the time sequence values $\left(i_{1},i_{2},i_{3},\;\ldots ,\;i_{\tau }\right)$, where τ is the last temporal data point in the sequence. The hidden states from this layer $\left(h_{i}^{1}\right)$ were sent to the second LSTM layer as inputs. The last hidden state from this layer $\left(h_{i}^{2}\right)$ corresponds to the input for the linear layer, as it stores information from all the sequences. Finally, the output $\left(y\right)$ was generated as the predicted label for the input sequence. An Adam optimizer was selected as the algorithm to compute the backpropagation through time (BPTT) problem [47] and Binary Cross-Entropy was selected as the loss function [41]. The training of the LSTM models included a validation method to minimize overfitting: The model performance was evaluated on the validation set and the loss function was computed. If the loss value was lower than the one evaluated in the previous step, then the latest network parameters were saved [48]. The number of hidden neurons per layer (20, 35 and 50) and the learning rate (0.005, 0.001 and 0.0001) were explored in order to find the optimal classification results.

### 2.4. Performance Metrics

Evaluation of the performance of the classification algorithms was conducted using five different metrics: precision, recall, specificity, accuracy and *f*_1_ score, according to the following equations, where *TP* represents true positive values, *TN* represents true negative values, *FP* represents false positive values and *FN* represents false negative values [41].

(1) $$Precision=\frac{TP}{TP+FP}$$

(2) $$Accuracy=\frac{TP+TN}{TP+FP+TN+FN}$$

(3) $$Recall=\frac{TP}{TP+FN}$$

(4) $$Specificity=\frac{TN}{TN+FP}$$

(5) $$f_{1}=2\cdot \frac{Precision\;\cdot Recall}{Precision+Recall}$$

## 3. Results

**Figure 3:**
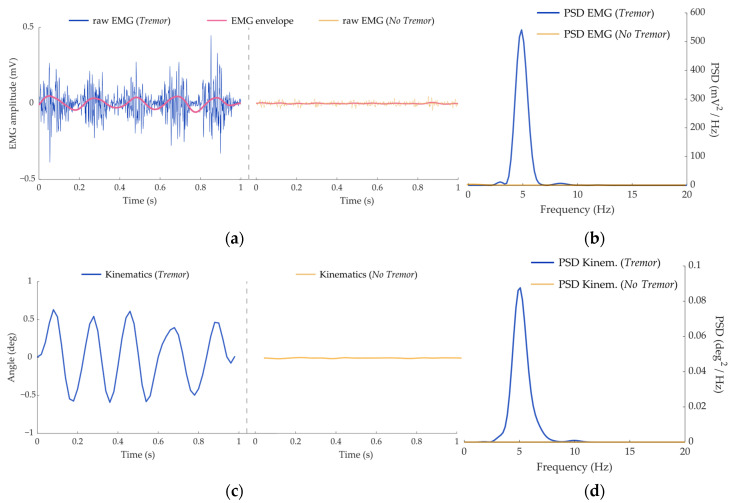
Illustrations of the signals contained in the three datasets. (**a**) Raw EMG signals from a tremor patient (blue line) and a healthy subject (yellow line). The estimated EMG envelopes (rose lines) were used as input signals in one of the three datasets. (**b**) Kinematic signals from a tremor patient (blue line) and a healthy subject (yellow line). (**c**) PSD function estimated for the EMG data displayed in (**a**). (**d**) PSD function estimated for the kinematics data displayed in (**b**).

Figure 3 illustrates examples of the kinematic and EMG signals used as inputs for the classifiers. The maximum peak of the PSD in the tremor band of each signal modality was used to label the datasets (Figure 3c,d) into *Tremor* and *No Tremor* classes. The examples depicted in Figure 3 correspond to clear manifestations of pathological tremors and non-pathological behaviors, which are reflected in the PSD functions. In both kinematic and EMG envelope signals, the tremor component can be visually identified as a sinusoidal waveform, whereas the raw EMG signal retains the oscillatory electrical muscle activity.

For each of the three datasets (kinematics, EMG envelope and raw EMG) and each classification technique, the optimal parameters for each classifier were found through the validation process (k-fold cross validation for the traditional machine learning methods, and training and validation process for the LSTM neural networks). Table 1 contains the parameters achieving the optimal classification results for each model and type of input data. The models that showed the best performance in the validation process were finally assessed with the training set.

**Figure 4:**
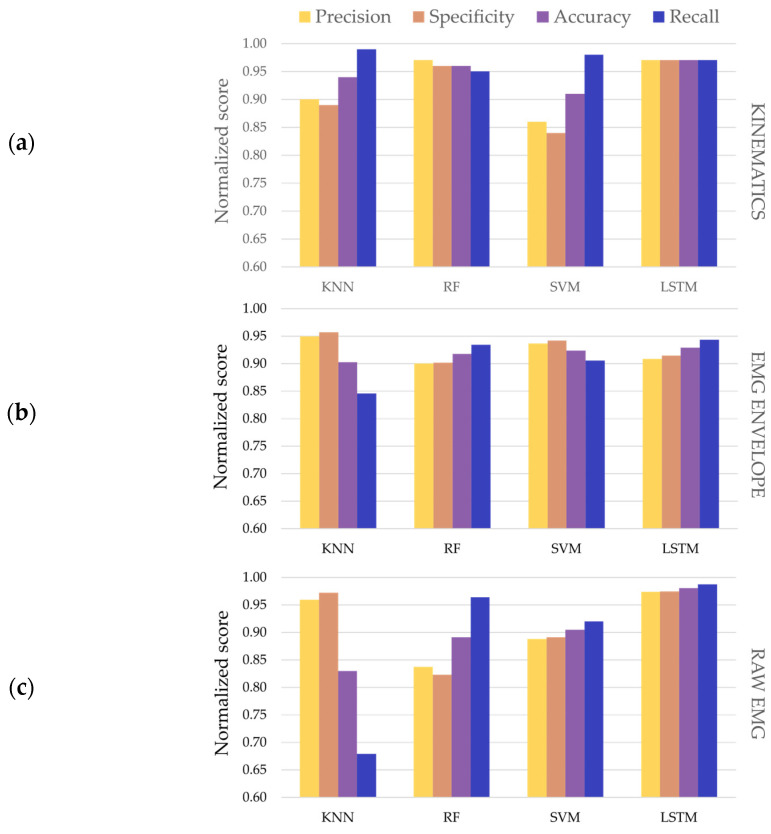
Classification results (precision, specificity, accuracy and recall) for the optimal classifiers and input data modalities: (**a**) Kinematics; (**b**) EMG envelope; (**c**) Raw EMG.

Figure 4 summarizes the classification results that were obtained for the optimal models and signal modalities. All the trained models obtained performance metrics above 0.80, except for the recall metric obtained by the KNN model using raw EMG as input data (0.64). For the kinematics data, all the models showed classification scores higher than 0.84 in all the assessment metrics. The highest value was achieved by the KNN model for the recall metric (0.99).

**Figure 5:**
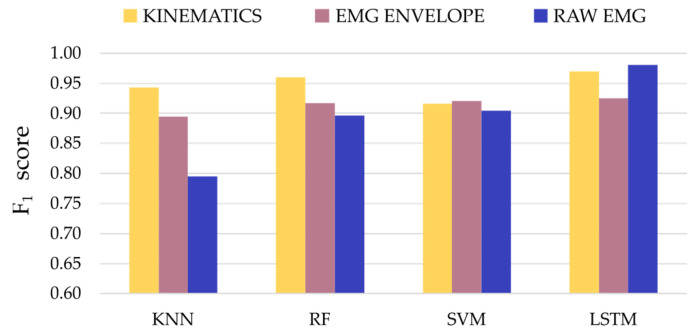
F_1_ scores for the optimal classifiers and input signal modalities.

When comparing the capability of the models to use different signal modalities to detect tremors, the f_1_ score was selected as it represents the harmonic mean between precision and recall (see Figure 5). The f_1_ score was higher than 0.80 for all the classifiers and types of input data. The performance of the KNN models in classifying the raw EMG data was lower compared to the rest of classifiers. All the LSTM models achieved the highest f_1_ scores for the different input datasets (f_1_-kinematics = 0.94, f_1_-EMG envelope = 0.96 and f_1_-EMG raw = 0.9) when compared to the rest of the classifiers. Additionally, the f_1_ scores across the three types of input signals showed that, on average, the LSTM models outperformed the rest of the traditional machine learning classifiers (mean ± standard deviation; f_1_-KNN = 0.88 ± 0.08, f_1_-RF = 0.92 ± 0.3, f_1_-SVM = 0.91 ± 0.01 and f_1_-LSTM = 0.96 ± 0.03). The KNN models showed the lowest average f_1_, whereas the RF and SVM models showed similar average performance.

Classification of the kinematic signals resulted in higher f_1_ scores compared to the classification of EMG envelope or raw EMG datasets for the same models used for the three traditional machine learning models. Nevertheless, the classification of raw EMG signals through the LSTM model was an exception to this, since it achieved the best classification f_1_ score (0.98). The averaged f_1_ scores across the four models for the three different datasets showed that the kinematics data resulted in more robust classification outcomes, followed by the pre-processed EMG (EMG envelope) and, lastly, the raw EMG (mean ± standard deviation; f_1_-kinematics = 0.95 ± 0.02, f_1_-EMG envelope = 0.91 ± 0.1 and f_1_-raw EMG = 0.89 ± 0.08).

## 4. Discussion

This study showed the feasibility of using machine learning techniques to provide binary classifications of pathological tremor signals without specific feature extraction. Three datasets containing kinematic, pre-processed (EMG envelopes) and raw EMG signals were used to train, validate and test different machine learning models in order to classify the input sequences into *Tremor* and *No Tremor* classes. The results showed high classification performances (f_1_ score above 0.8) for all the models and data input modalities. Moreover, the LSTM neural networks achieved, on average, the best classification outcomes for all the datasets, including the classification of raw EMG signals.

Overall, the different models provided high classification outcomes. The KNN models showed the highest variability, ranging from 0.64 to 0.99 in the recall metric. Moreover, it achieved the lowest f_1_ score among the classifiers (0.8) for the classification of raw EMG signals. No significant differences were found between the RF and SVM classifiers. The LSTM models achieved, on average, the best performance scores for the different modalities of input data compared to the traditional models, verifying the authors’ hypothesis. The higher complexity of the LSTM neural networks when extracting temporal patterns, such as the tremor cycles, could explain the difference in performance when compared to the traditional methods. Nevertheless, this difference was subtle for the kinematics and EMG envelope datasets. Thus, the application of more complex models does not necessarily lead to better outcomes. In these scenarios, other factors, such as the computational cost throughout the training and execution processes, should be considered when selecting one of the models.

Since a minimum pre-processing stage was applied in order to isolate the tremorgenic component between 3 and 10 Hz for both kinematic and EMG envelope signals, the high signal-to-noise ratio in the frequency band was expected to result in better classification results. Therefore, when comparing signal input modalities, it was expected that the classification of the kinematic signals would lead to the highest classification scores, followed by the EMG envelope dataset, and finally the raw EMG signals. This behavior was confirmed by the traditional classifiers, which achieved higher f_1_ scores for the kinematic signals, then for the EMG envelope and finally for the raw EMG dataset. Interestingly, the LSTM model achieved the highest f_1_ score (0.98) for the classification of raw EMG signals. This result highlights the capability of LSTM networks as a deep learning tool to extract temporal patterns from un-processed signals.

No direct comparison between the results of this study and other research from the scientific literature could be found, as both the datasets and the approach followed in this study were unique, to the best of our knowledge. Interestingly, another group has reported f_1_ classification scores ranging between 0.64 and 0.80 for different parametric and non-parametric signal processing methods used to classify signals containing 4-s acceleration data from patients diagnosed with pathological tremors of the hand (compared to classifications performed through visual inspection by trained neurologists) [49]. In another study, the envelopes in the tremor band of acceleration and EMG signals recorded from ET, PD and healthy participants were used to develop a tremor binary classifier based on the PSD and a thresholding method. The results showed accuracy scores (when compared to visual classification) for the acceleration dataset of 0.90 and 0.94 for the EMG envelope dataset [50]. These examples support the validation of the classifiers obtained in this study, where shorter sequences of data (including unprocessed EMG signals) were used to classify tremors with f_1_ scores above 0.9 for different sorts of input modalities when compared against traditional PSD-thresholding methods.

Both traditional implementations and deep learning models were capable of detecting the tremor pattern from the time series without any previous information of the input signals. This approach is relevant because there was no pre-processing of the raw data to extract any signal features, such as descriptive statistics, time-frequency or signal power measurements [25,45]. Though pathological tremor signals are variable and non-stationary in short periods of time, both the kinematic and EMG recordings exhibited tremor patterns which could be differentiated from non-pathological behaviors. Interestingly, the raw EMG signals from healthy and ET patients could contain some oscillatory components related to physiological (non-pathological) tremors between 8 Hz and 12 Hz [5]. Despite this oscillatory pattern, all the classification models, particularly the LSTM models, could disregard this pattern, which is characterized by a different frequency and amplitude compared to those seen in pathological tremors.

The capability of these models to classify specific tremor patterns that are exclusively related to ET and that are different from other oscillations caused by different neural disorders (e.g., PD or dystonia) was not explored in this study. Diagnosis outcomes based on the classification of kinematics or EMG data could provide additional value to the current clinical diagnosis, which is based on clinical scales, as other research groups have shown [51]. Datasets including EMG and/or kinematic signals recorded from different motor tasks than the ones used in this study (postural or isometric contractions) would be necessary in order to collect a sufficient sample of tremor patterns associated with different etiologies [52].

PES systems to reduce tremors could benefit from these implementations to guide the stimulation if a tremor is detected [14]. Following this procedure, the length of the signal processing pipeline would be shortened compared to other approaches that use multiple feature extraction [26]. Translation from computational models to embedded systems would require a transition from offline to online implementations, a process which might be challenging. Due to the size of the data used as input for the classifiers, ranging from 50 samples per second (for the EMG envelopes and kinematics) to 510 samples per second (for the raw EMG), the computing time for the classification of one instance is estimated to be in the order of milliseconds. Following the assumption that the computing time of the different algorithms does not have a significant impact on real-time performance, the main criteria used to select one of the algorithms would rely on the optimal classification outcomes for each specific modality of input data. In future studies, it would be valuable to explore different input sequence lengths in order to provide higher time resolution, as well as the optimization of model parameters in order to meet the real-time requirements, and finally to test the feasibility of these models in a real-time system.

## Figures and Tables

**Table 1:** Model parameters used for the optimal classifiers selected for the final assessment.

KNN	Algorithm	Leaf Size	Metric	Neighbors	Weights
Kinematics	Ball-Tree		Euclidean	6	Distance
EMG envelope	Ball-Tree	30	Euclidean	3	Distance
EMG Raw	Ball-Tree	30	Chebyshev	2	Distance
**RF**	**Criterion**	**Max. Features**	**Min. Samples**	**Trees**	
Kinematics	Entropy	sqrt	2	100	
EMG envelope	Entropy	sqrt	2	100	
EMG Raw	Entropy	sqrt	2	110	
**SVM**	**C**	**γ**	**Kernel**		
Kinematics	10	1	RBF		
EMG envelope	1	0.1	RBF		
EMG Raw	10	0.01	RBF		
**LSTM**	**Learning Rate**	**Hidden Size**			
Kinematics	0.005	50			
EMG envelope	0.005	35			
EMG Raw	0.005	50			

## Data Availability

The datasets used and/or analyzed during the current study are available from the corresponding author on reasonable request.
